# RA Acts in a Coherent Feed-Forward Mechanism with *Tbx5* to Control Limb Bud Induction and Initiation

**DOI:** 10.1016/j.celrep.2015.06.068

**Published:** 2015-07-23

**Authors:** Satoko Nishimoto, Susan M. Wilde, Sophie Wood, Malcolm P.O. Logan

**Affiliations:** 1 Randall Division of Cell and Molecular Biophysics, Guy’s Campus, King’s College London, London SE1 1UL, UK; 2 Procedural Services Section, MRC-National Institute for Medical Research, The Ridgeway, Mill Hill, London NW7 1AA, UK

## Abstract

The retinoic acid (RA)- and β-catenin-signaling pathways regulate limb bud induction and initiation; however, their mechanisms of action are not understood and have been disputed. We demonstrate that both pathways are essential and that RA and β-catenin/TCF/LEF signaling act cooperatively with Hox gene inputs to directly regulate *Tbx5* expression. Furthermore, in contrast to previous models, we show that *Tbx5* and *Tbx4* expression in forelimb and hindlimb, respectively, are not sufficient for limb outgrowth and that input from RA is required. Collectively, our data indicate that RA signaling and Tbx genes act in a coherent feed-forward loop to regulate *Fgf10* expression and, as a result, establish a positive feedback loop of FGF signaling between the limb mesenchyme and ectoderm. Our results incorporate RA-, β-catenin/TCF/LEF-, and FGF-signaling pathways into a regulatory network acting to recruit cells of the embryo flank to become limb precursors.

## Highlights

• RA and β-catenin signaling directly regulate *Tbx5* expression in forelimb induction
• Input from RA is required for hindlimb induction and initiation
• *Tbx5* and *Tbx4* in forelimb and hindlimb are not sufficient for limb initiation
• RA and Tbx genes act in a coherent feed-forward loop to regulate *Fgf10* expression

## Graphical Abstract

**Figure:**
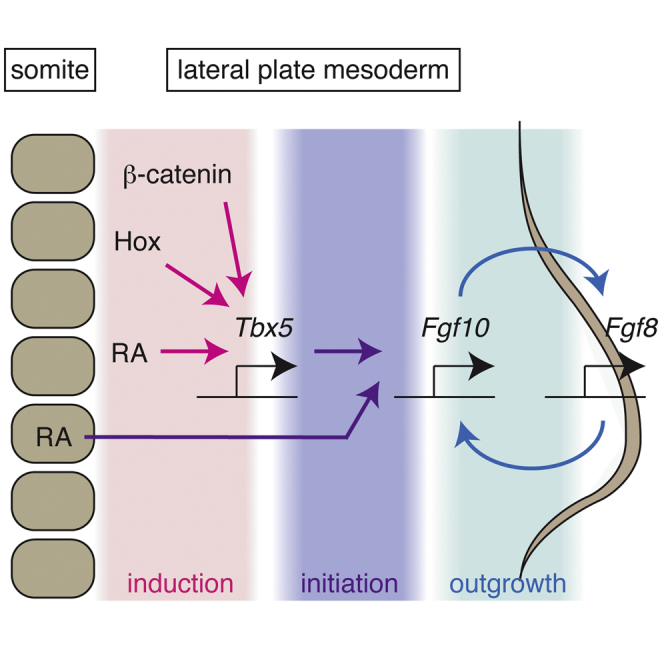

## Teaser

Nishimoto et al. reveal the regulatory network including retinoic acid (RA), β-catenin pathway, and Tbx genes controlling the early steps of limb formation. This study demonstrates the shared role of RA in forelimb and hindlimb and an evolutionarily conserved role of the β-catenin pathway in limb induction.

## Introduction

Limb bud outgrowth is initiated and maintained by establishing a positive feedback loop of FGF signaling comprised of *Fgf10* expressed in the lateral plate mesoderm (LPM), inducing the expression of *Fgf8* in the overlying, distal ectoderm (Boulet et al., 2004; Min et al., 1998; Ohuchi et al., 1997; Sekine et al., 1999; Xu et al., 1998). Initial expression of *Fgf10* in the forelimb- and hindlimb-forming LPM is controlled by Tbx transcription factors, Tbx5 in the forelimb and Tbx4 in the hindlimb (Duboc and Logan, 2011), and deletion of either *Tbx5* or *Tbx4* causes outgrowth defects of limb buds (Agarwal et al., 2003; Naiche and Papaioannou, 2003; Ng et al., 2002; Rallis et al., 2003; Takeuchi et al., 2003). In addition, a recent study showed *Tbx5* and *Fgf10* are required for limb progenitor cells to undergo an epithelial-to-mesenchymal transition (Gros and Tabin, 2014). However, the regulatory mechanisms that control activation of *Tbx5* and *Tbx4* expression and how these genes regulate *Fgf10* expression are not understood.

Classical embryological experiments in the chick established that an inductive interaction between the paraxial mesoderm and the LPM is required for limb bud formation. Insertion of an impermeable barrier between the somites and the adjacent LPM at forelimb or hindlimb level in a chick embryo at stages 13–16 blocks limb bud outgrowth (Murillo-Ferrol, 1965; Stephens and McNulty, 1981; Sweeney and Watterson, 1969). If, however, a permeable barrier is used, limbs of normal morphology but smaller size form. Furthermore, somites, but not intermediate mesoderm, have the ability to induce ectopic limb buds from forelimb or hindlimb, forming LPM explants when grafted into a non-limb region of the flank or coelomic cavity (Kieny, 1969; Pinot, 1970).

There is evidence the inductive signal from the paraxial mesoderm essential for forelimb bud initiation is retinoic acid (RA). Zebrafish embryos mutant for the gene *retinaldehyde dehydrogenase-2* (*Raldh2*), an enzyme that oxidizes retinal to RA, lack pectoral fins (Begemann et al., 2001; Grandel et al., 2002), the homologous structure to the forelimb in amniotes. Genetic ablation of somitic mesoderm leads to reduced *Tbx5* expression, and this effect can be rescued by exogenous RA, suggesting the somitic mesoderm is the source of RA in this process (Gibert et al., 2006). A requirement for RA signaling in the initiation of limb outgrowth in tetrapods has also been demonstrated (Niederreither et al., 1999; Stratford et al., 1996). Inhibition of RA synthesis by disulphiram abolishes forelimb outgrowth in chick embryos (Stratford et al., 1996). In mouse embryos, deletion of *Raldh2* arrests development around E8.5–8.75 and forelimb buds are not formed (Niederreither et al., 1999). To extend embryo survival, RA was maternally administered (Mic et al., 2002, 2004; Niederreither et al., 2002) and the rescued embryos show smaller forelimb buds, whereas the hindlimb buds are normal. As *Raldh3* is expressed in the mesonephros adjoining the hindlimb buds, similar rescue experiments were performed with *Raldh2/Raldh3* double mutants. The hindimb buds of the rescued *Raldh2/Raldh3* mutants are normal, which has been interpreted as demonstrating that RA is not required for hindlimb outgrowth (Zhao et al., 2009). This interpretation has been contested on the basis that it is difficult to exclude the possibility that RA administered to the mother has not had some impact on limb formation in the embryos (Roselló-Díez et al., 2014).

Evidence from a number of models indicates that the Wnt-signaling pathway acts upstream of *Tbx5* in both zebrafish and chick embryos (Ng et al., 2002). In zebrafish, *Wnt2b* is expressed in tissue medial to the LPM at stages just prior to appearance of the pectoral fin buds. In chick embryos, *Wnt2b* is expressed in the medial sides of the embryonic coelom as well as in the somites (Kawakami et al., 2001). In both species, blocking of the Wnt pathway, using *Wnt2b* morpholino in zebrafish and using an adenovirus expressing *Axin* in chick, downregulates *Tbx5* (Ng et al., 2002). The role of the Wnt pathway in mouse limb initiation is less clear however. Expression of a candidate Wnt ligand in limb-forming LPM or adjacent tissues has not been reported. Furthermore, in embryos of mice mutant for the two TCF/LEF genes expressed in the limb, *Lef1* and *Tcf1*, limb buds form, although subsequent outgrowth is blocked consistent with an essential role of Wnt in AER maturation after initiation of a limb bud (Galceran et al., 1999). β*-catenin* conditional mutant mice do not form hindlimbs, suggesting that β*-catenin* is required for hindlimb initiation (Kawakami et al., 2011). The role of β-*catenin* in forelimb initiation, however, has not been studied in detail. Because studies of the regulation of Tbx genes by the Wnt pathway in zebrafish and chick were focused on pectoral fin and forelimb initiation, respectively, these studies do not address whether the role of the Wnt pathway in forelimb initiation is also conserved in mouse or how the Wnt pathway converges on other known pathways regulating limb formation.

In this study, we resolve some long-standing areas of confusion regarding the role of RA and β-catenin signaling and provide a molecular mechanism that explains and unifies previously conflicting reports. We show that RA and *Tbx* genes act in a coherent feed-forward loop controlling limb formation. At limb induction stages, an RA signal directly induces *Tbx* gene expression in the limb forming LPM together with β-catenin/TCF/LEF and Hox genes. Insertion of a barrier between the paraxial mesoderm and the LPM prevents *Tbx5* or *Tbx4* expression in the LPM, and *Tbx5* expression is restored by application of RA. In the hindlimb, *Tbx4* expression is downregulated by an RAR inverse agonist. Furthermore, we identify RA response elements (RAREs) and TCF/LEF-binding site in the *Tbx5* forelimb regulatory element and demonstrate their requirement for the activity of this regulatory sequence. Subsequently, at limb initiation stages, RA acts cooperatively with Tbx5 and Tbx4, potentially as a co-factor, to activate *Fgf10* transcription and as a result establishes the Fgf10-Fgf8 positive-feedback loop required for limb outgrowth. Barrier insertion after limb induction stages inhibits *Fgf10* and *Fgf8* expression and blocks limb outgrowth without affecting *Tbx5* or *Tbx4* expression. These defects are rescued by addition of exogenous RA. Furthermore, application of an RAR inverse agonist causes a reduction in *Fgf10* expression and results in smaller hindlimb buds. These results suggest that input from RA is essential for Tbx5 and Tbx4 to regulate *Fgf10* expression in the LPM. Our study reveals distinct molecular networks regulating two key steps of limb formation, limb induction and limb initiation, and provides a molecular framework to distinguish these two events.

## Results

### *Tbx5* or *Tbx4* Expression in the LPM Is Not Sufficient to Initiate Limb Outgrowth

**Figure 1:**
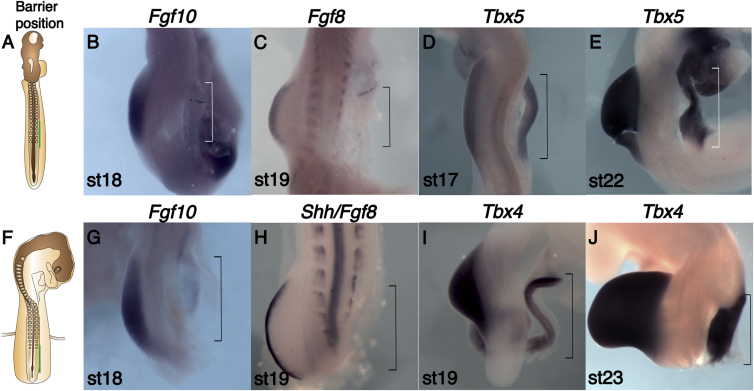
Marker Gene Expression Changes following Barrier Insertion at the Wing- and Leg-Forming Regions (A) Stage 13 chick embryo schematic showing barrier position (green line) between somites and LPM at the presumptive wing level (somites 15–20). (B–E) WISH analysis on operated embryos. The wing region is outlined in brackets and stage indicated. (B) *Fgf10* expression is detected in the unoperated bud but absent on the operated side. (C) *Fgf8* expression is absent on the operated side, suggesting absence of AER. (D) *Tbx5* expression is present in the operated LPM at the same level as the control left wing bud. (E) *Tbx5* is expressed in the operated wing region despite absence of limb growth. (F) Schematic showing barrier position at the presumptive leg level (somites 26–32) in stage 15 embryos. (G–J) WISH analysis on operated embryos. The leg region is outlined (brackets). (G) *Fgf10* expression is absent in the operated LPM, but robust expression is detected on the left bud. (H) *Fgf8* expression is absent in the operated side, suggesting there is no AER. (I) *Tbx4* is expressed in the LPM at the same rostro-caudal level as the control bud. (J) *Tbx4* expression is maintained in the right leg region despite absence of limb growth.

Studies in chick and mice have demonstrated *Tbx5* is required in the LPM for initiation of forelimb bud outgrowth and that a critical function of *Tbx5* is to activate expression of *Fgf10*, which is essential for limb outgrowth (Agarwal et al., 2003; Ng et al., 2002; Rallis et al., 2003). Other studies in the chick have suggested that *Tbx5* is sufficient to induce limb formation (Ng et al., 2002; Takeuchi et al., 2003). To study these issues further, we placed impermeable foil barriers between the forming somites and the LPM at the level of somites 15–20 at stages 12 to 13 (Figure 1A) before *Tbx5* and *Fgf10* are expressed in the LPM. Following this operation, the foil barrier was opposite the contralateral wing bud by stages 16 to 17, but there was no outgrowth of the LPM distal to the barrier on the operated side of the embryo and no AER in the ectoderm (Figures 1B–1E; Table S1). Forty percent of these operations led to the death of the embryo (Table S1), probably due to the proximity of the incision to major blood vessels. When analyzed by whole-mount in situ hybridization, expression of *Fgf10* (Figure 1B) and *Fgf8* (Figure 1C) were not detected on the operated side of the forelimb-forming region of the LPM, whereas robust expression was obvious on the contralateral control side, consistent with complete absence of the forelimb bud and failure to establish an AER. Surprisingly, however, when operated embryos were analyzed for *Tbx5*, robust expression was detected on the operated side with no apparent effects on the levels or the extent of expression (Figure 1D). Even when operated embryos were allowed to develop until stage 22, robust expression of *Tbx5* was detected in the forelimb-forming LPM despite the failure of these cells to form a limb (Figure 1E).

To test whether *Tbx4* expression in the hindlimb-forming region follows a similar pattern following insertion of a barrier, we positioned foil barriers between the somite-forming segmental plate (at the approximate level of somites 26–32) and the LPM at stage 15 (Figure 1F), as previously described (Murillo-Ferrol, 1965). Equivalent to what was observed following the wing level operation, by stages 18 to 19, the barrier was found opposite the leg bud on the contralateral side of the embryo and there was no leg or AER formation distal to the barrier (Figures 1G–1I; Table S2). Although *Fgf10* and *Fgf8* expression were not detected in hindlimb-level LPM (Figures 1G and 1H), *Tbx4* was expressed distal to the barrier at the same rostro-caudal level as the control contralateral side (Figure 1I) and this domain of expression was still detected in operated embryos harvested at stage 23 (Figure 1J). Another marker of hindlimb mesenchyme, *Pitx1*, was also expressed in the LPM distal to the barrier in a similar way to *Tbx4* (Figures S1B and S1C). Together, our results indicate *Tbx5* and *Tbx4* expression domains can be established following barrier placement at these stages, neither *Tbx5* (forelimb) nor *Tbx4* (hindlimb) are sufficient for *Fgf10* expression, and additional signals from axial tissues are necessary to establish *Fgf10* expression in the limb-forming LPM.

### RA from the Somites Is Essential in the LPM before *Fgf10* Induces Limb Bud Outgrowth

*Fgf10* mouse knockout has demonstrated the gene is required for both forelimbs and hindlimbs to develop from the embryo flank (Min et al., 1998; Sekine et al., 1999; Xu et al., 1998). In the chick, application of exogenous Fgfs can induce ectopic limb formation from the inter-limb flank (Cohn et al., 1995, 1997; Ohuchi et al., 1997). We tested whether application of an FGF-soaked bead into the LPM is able to rescue limb formation following barrier insertion.

**Figure 2:**
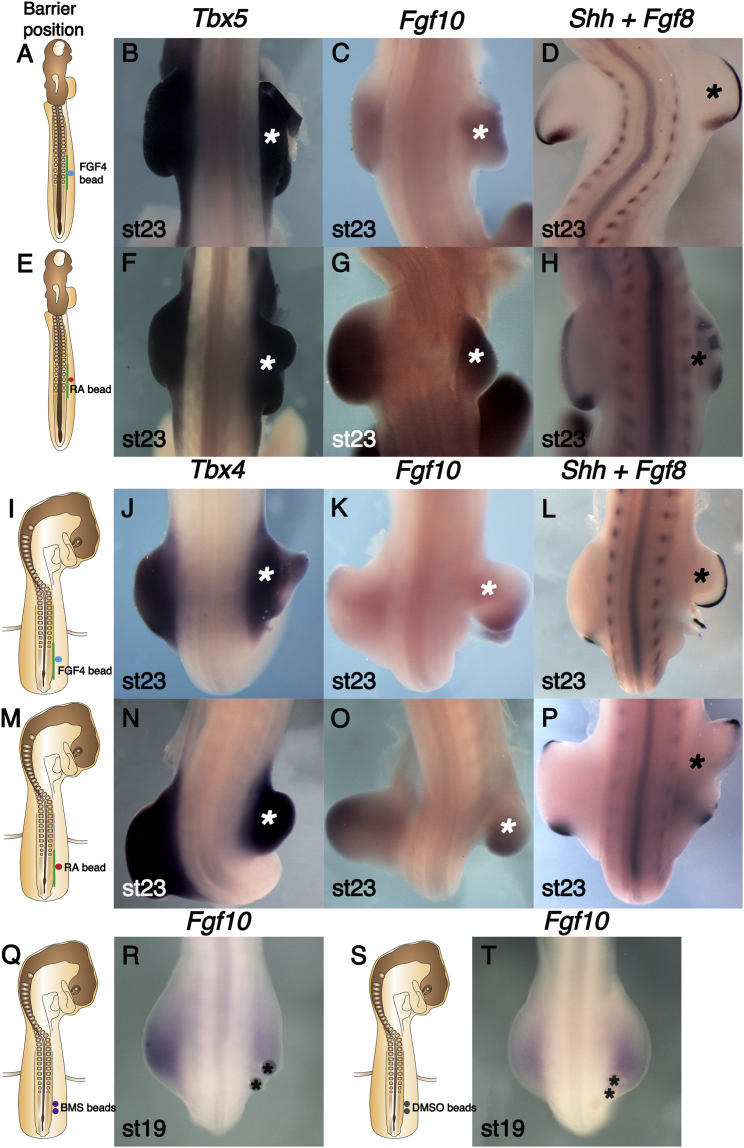
RA Rescues Limb Buds Absence Caused by Barrier Insertion (A) Stage 13 chick embryo schematic showing barrier position (green line) between somites and LPM at the presumptive wing level (somites 15–20) and FGF4-soaked beads (blue circle). (B–D) FGF4 rescues wing bud outgrowth (shown by asterisk). (B) *Tbx5* expression is present in the rescued wing bud similar to the un-operated left side. (C) *Fgf10* expression in the rescued wing bud is similar to the un-operated left side. (D) *Fgf8* expression in the AER and *Shh* at the posterior margin are established in the rescued wing bud. (E) The same schematic as in (A), indicating where RA bead (red circle) was placed prior to barrier insertion. (F–H) RA rescues wing outgrowth (shown by asterisk). (F) *Tbx5* expression is present in the rescued right wing bud. (G) *Fgf10* expression in the rescued wing bud is similar to the un-operated side. (H) *Fgf8* is expressed in the AER of the rescued wing bud. (I) Schematic diagram indicating barrier position (green line) at the presumptive leg level (somites 26–32) and an FGF4-soaked bead (blue circle). (J) *Tbx4* expression in the rescued right leg bud in a similar pattern to that in the un-operated left side. (K) *Fgf10* expression in the rescued leg bud is similar to the un-operated left side. (L) *Fgf8* is expressed in the AER of the rescued leg bud. (M) The same schematic as in (I), indicating where an RA bead (red circle) was placed. (N) *Tbx4* expression is present in the rescued leg bud similar to the un-operated side. (O) *Fgf10* is expressed in the rescued leg bud similar to the un-operated side. (P) *Fgf8* is expressed in the AER of the rescued right bud. (Q) Schematic indicating where BMS493 beads (purple circles) were placed. (R) *Fgf10* expression is downregulated on the operated right side. Beads are asterisked. (S) Similar schematic as (Q), indicating the position of control DMSO beads (gray circles). (T) DMSO beads (shown by asterisks) did not affect *Fgf10* expression.

Foil barriers were placed at the prospective wing level in stage 13 chick embryos (as shown in Figure 1A), and in addition, a bead soaked in FGF4 was inserted into the LPM distal to the barrier (Figure 2A). Wing buds of a near normal morphology emerged distal to the barrier following application of an FGF-soaked bead (Figures 2B–2D; Table S1). *Tbx5*, *Fgf10*, *Fgf8*, and *Shh* were all expressed in their normal patterns in rescued wing buds. Therefore, experimental addition of FGF to the LPM can support limb bud and AER formation when signals from axial tissues are blocked by a barrier.

Studies of *Raldh2* mutants demonstrate that RA signaling is essential prior to forelimb initiation in mouse and zebrafish embryos (Gibert et al., 2006; Grandel et al., 2002; Niederreither et al., 1999). RA has also been shown to be required in the chick LPM for limb outgrowth (Stratford et al., 1996). Crucially, cells from wild-type zebrafish somites are able to rescue pectoral fin bud initiation in *Raldh2* mutant fish (Gibert et al., 2006), suggesting a requirement for RA from the somites before forelimb bud initiation in zebrafish. We tested whether the axial signal blocked by a barrier is RA by placing an RA-soaked bead distal to a prospective wing level barrier (Figure 2E). Following this operation, wing bud outgrowth occurred and *Tbx5*, *Fgf10*, *Fgf8*, and *Shh* were all expressed in the rescued buds (Figures 2F–2H; Table S1). The RA-rescued buds were occasionally bifurcated with an apparent gap in the AER at the indentation site. In control experiments, wing buds were absent adjacent to barriers that had a control DMSO-soaked bead placed in the LPM (Table S1).

As *Raldh2* is expressed in the forelimb LPM at limb initiation stages, we tested whether barrier insertion causes limb outgrowth defects by downregulating *Raldh2* in the LPM. *Raldh2* was expressed in the LPM distal to the barrier at a similar expression level as that of the control left side (Figure S2), suggesting that barrier insertion does not affect local RA production in the LPM. This result indicates that the activity of RA produced in the LPM is not sufficient to induce *Fgf10* expression and that RA from axial tissues is essential.

To test whether there is a similar requirement for RA in presumptive leg bud LPM, we carried out similar bead experiments with prospective leg-level barriers placed at stage 15 (Figures 2I and 2M). Application of FGF4-soaked beads rescued leg bud outgrowth, and *Tbx4*, *Fgf10*, and *Fgf8* were expressed in the rescued buds (Figures 2J–2L; Table S2). Implanted RA-soaked beads were also able to rescue hindlimb formation, and *Tbx4*, *Fgf10*, and *Fgf8* were expressed in the rescued leg buds (Figures 2N–2P; Table S2). In some instances, the rescued buds had a bifurcated morphology with a medial gap in the AER (Figure 2P). This was also seen in RA-rescued wing buds and may be due to the RA dose used being too high because the AER can degenerate in the presence of high RA concentrations (Lee and Tickle, 1985; Tickle et al., 1989). These results demonstrate that, if signals from axial tissues are blocked, a source of RA applied to the LPM can rescue wing and leg formation.

To confirm the requirement for RA in hindlimb initiation, we used an inverse agonist of RAR, BMS 493. *Fgf10* expression was downregulated following application of BMS 493 beads in the LPM, resulting in smaller hindlimb buds compared to the control side (Figures 2Q and 2R; Table S3). Control DMSO beads did not cause these defects (Figures 2S and 2T; Table S3), demonstrating the specific effects of BMS 493 and to exclude the possibility that these defects were caused by mechanical damage caused by insertion of beads. The defects observed following BMS 493 application are milder than those induced by barrier operation, e.g., *Fgf10* is still expressed and limb buds are formed. This is likely because the effects of BMS 493 are restricted locally around the beads and may not be able to antagonize all the RA produced in axial tissues. Together, these results suggest that an RA signal from the axial tissues is essential for *Fgf10* to be expressed in limb bud.

### Early Axial Signals Specify the LPM Cells that Later Express *Tbx5* or *Tbx4*

**Figure 3:**
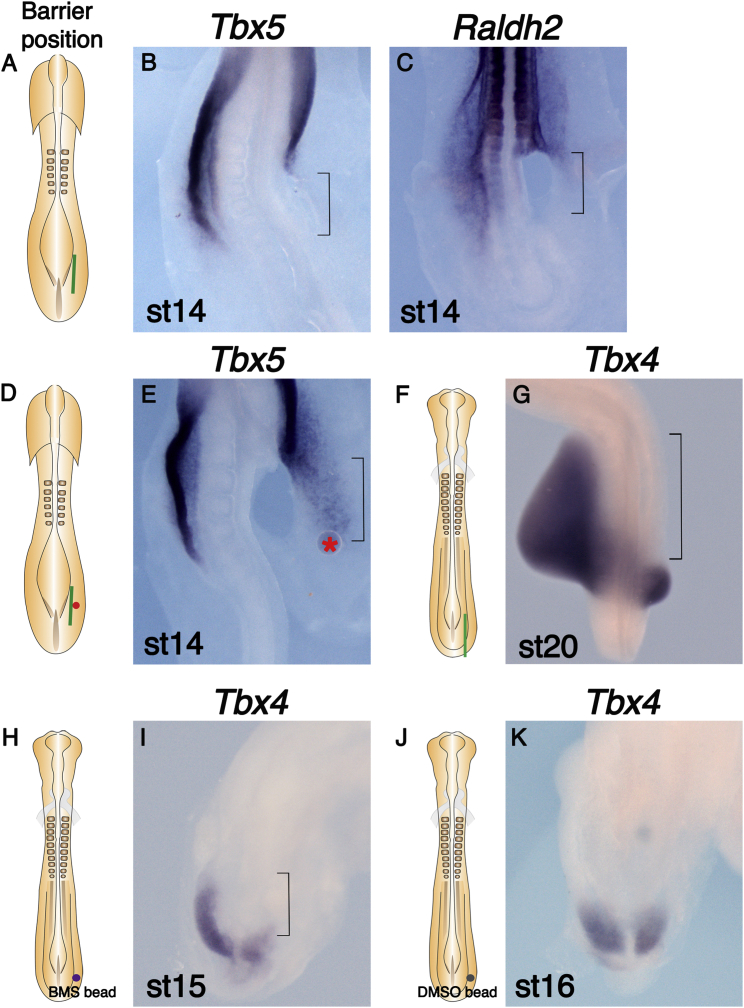
Downregulation of *Tbx5* and *Tbx4* Expression following Early Stage Barrier Insertion (A) Schematic diagram of a stage 9 chick embryo indicating barrier position (green line) between the presomitic mesoderm and LPM at the presumptive wing level. (B and C) WISH carried out on operated embryos. (B) *Tbx5* expression is absent in the operated LPM (indicated by bracket). (C) *Raldh2* expression is downregulated on the operated side (indicated by bracket). (D) Schematic diagram of a stage 9 embryo showing the positions of a barrier (green line) and RA bead (red circle). (E) *Tbx5* expression is rescued (indicated by bracket) by an RA bead (red asterisk). (F) Schematic diagram of a stage 10 chick embryo showing barrier position (green line) at the presumptive leg level. (G) *Tbx4* expression is absent in the operated right LPM (bracket). (H) Schematic diagram showing BMS 493 bead position (purple circle). (I) *Tbx4* expression is downregulated on the operated side (bracket). (J) Schematic diagram showing control DMSO bead position (gray circle). (K) *Tbx4* is expressed on the operated right side at the similar level to that of control left side.

We tested when the LPM acquires its ability to express *Tbx5* or *Tbx4* in the prospective wing- and leg-forming regions, respectively. When barriers were inserted between the paraxial mesoderm and the LPM proximal to the presumptive wing at stages 8 to 9, wing bud outgrowth was blocked (Table S1). In contrast to results from barrier placement at stage 13, *Tbx5* expression was absent in the forelimb-forming LPM (Figures 3A and 3B). These results demonstrate that an axial signal at stages 8 to 9 is required for the adjacent LPM cells to express *Tbx5* at the correct rostro-caudal level at stage 14.

Next, we tested whether RA can rescue *Tbx5* expression inhibited by this early barrier, as we demonstrated that the RA signal can rescue the limbless phenotype caused by barrier insertion at later stages (Figures 2F–2H and 2N–2P). *Raldh2* is expressed in the presumptive forelimb prior to *Tbx5* expression (Swindell et al., 1999). Following barrier insertion, *Raldh2* expression in the wing level LPM of a stage 13 to 14 embryo is downregulated compared to the control left side (Figure 3C), suggesting that a signal from axial tissues is required to induce *Raldh2* and supply RA locally in the LPM. We attempted to rescue *Tbx5* expression in the LPM following insertion of an early barrier by adding RA-soaked beads (Figure 3D). By stage 14, when *Tbx5* expression is first detected, *Tbx5* expression distal to the barrier was rescued (compare Figure 3E to Figure 3B).

We also developed a protocol to place barriers opposite the leg bud to investigate effects on *Tbx4* expression. Barriers inserted between the paraxial mesoderm and the presumptive leg LPM at stage 10 blocked leg bud formation and expression of *Tbx4* in the leg-forming LPM (Figures 3F and 3G), suggesting that a signal from axial tissues at stage 10 is required for later expression of *Tbx4* in hindlimb LPM. Furthermore, we tested whether the inverse agonist of RAR reduces *Tbx4* expression. A BMS 493 bead placed in the hindlimb LPM at stage 10 downregulates *Tbx4* (Figures 3H and 3I), whereas control beads soaked in DMSO did not affect *Tbx4* expression (Figures 3J and 3K).

Together, these results support a model that an RA signal regulates limb induction by positively regulating *Tbx5* and *Tbx4* in the forelimb and hindlimb LPM, respectively.

### RA Signal Directly Regulates *Tbx5* Expression

**Figure 4:**
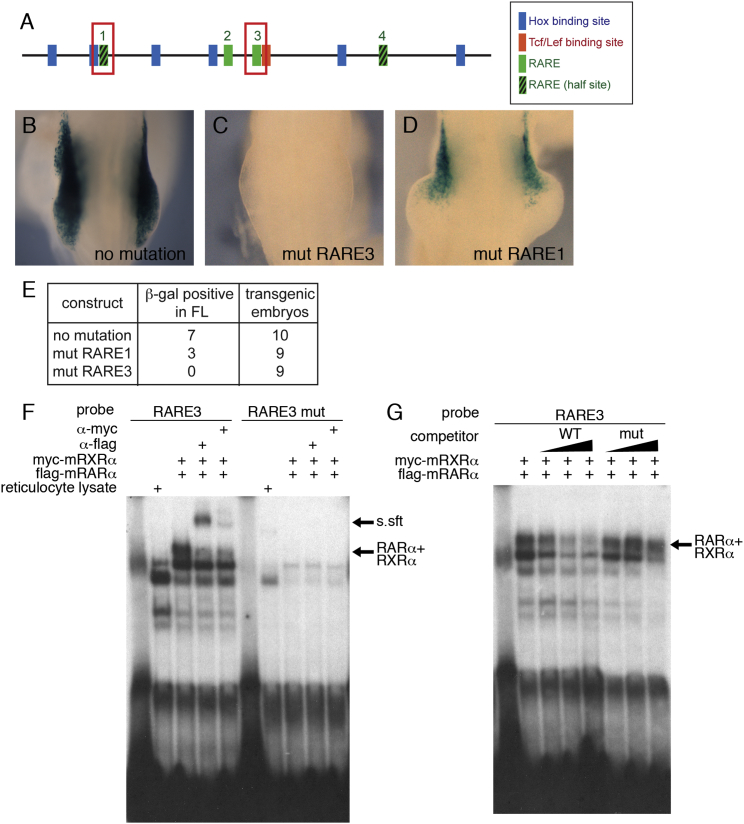
RAREs in the *Tbx5*-Regulatory Element Are Required for Its Enhancer Activity (A) Schematic representation of the mouse *Tbx5* forelimb-regulatory element. This 361-bp sequence contains six Hox-binding sites (blue boxes), one TCF/LEF site (red box), and two RAREs and two RARE half-sites (green boxes). (B–D) Transient transgenic mouse embryos at E9.0–9.5. Embryos were stained for β-galactosidase for wild-type (B) or mutated (C and D) constructs of *Tbx5*int2(361) reporter. (B) The reporter expresses throughout the forelimb bud similar to endogenous *Tbx5* expression. (C) Mutation of RARE3 kills enhancer element activity. (D) Mutation of RARE1 downregulates enhancer activity. (E) Numbers of embryos showing the staining in the forelimb buds. (F) EMSA assay using in-vitro-translated flag-RARα (2 μl) and myc-RXRα (2 μl) proteins. RARα and RXRα form a complex with RARE3 probe (lanes 1–5), but not with RARE3 mutated probe (lanes 6–10). α-flag and α-myc antibodies (2 μl) super-shifted the complex (lanes 4 and 5). (G) Competition assays were carried out with excess amounts of unlabeled oligonucleotides (20×, lanes 3 and 6; 50×, lanes 4 and 7; 200×, lanes 5 and 8). RARE3 WT non-labeled competitor (lanes 3–5), but not RARE3-mutated competitor (lanes 6–8), abolished the RARα-RXRα-oligo complex, confirming the specificity of the complex.

**Figure 5:**
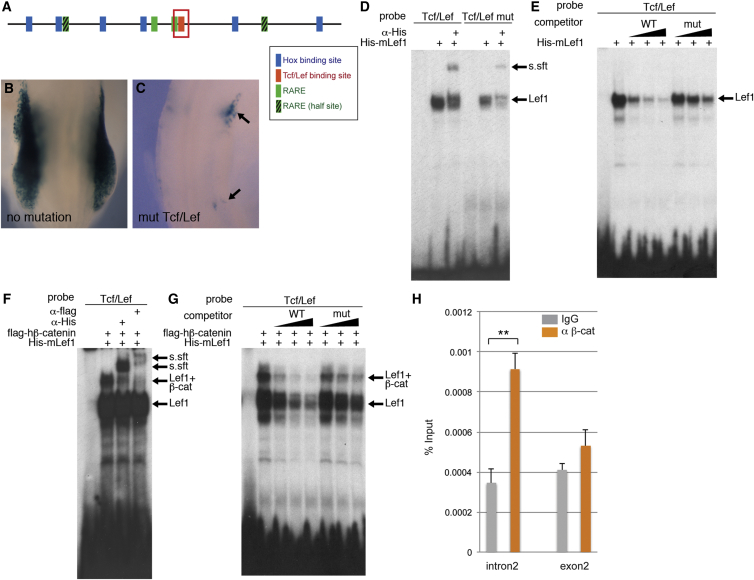
The TCF/LEF Site in the *Tbx5* Forelimb Enhancer Is Required for Its Activation in the Forelimb Bud (A) Schematic representation of the mouse *Tbx5*-regulatory element. A putative TCF/LEF site is shown in red. (B and C) E9.0–9.5 embryos were stained for β-galactosidase for wild-type *Tbx5*int2(361) reporter construct (B) or constructs with mutations on TCF/LEF site (C). (D) Binding of in-vitro-translated His-tagged mouse Lef1 protein to the TCF/LEF site. Lef1 (1 μl) can form a complex with an oligo probe containing the TCF/LEF site (TCF/LEF probe; lane 2). Specificity is confirmed by super-shift of the complex with α-His antibody (2 μl; lane 3). Lef1 weakly binds a mutated TCF/LEF probe (TCF/LEF mut probe; lanes 4–6). (E) Addition of non-labeled competitor abolishes the Lef1 protein-TCF/LEF oligo complex (lanes 2–5). TCF/LEF mut oligo weakly competes with labeled TCF/LEF probe (lanes 6–8). Excess amounts of unlabeled oligonucleotides were used (10×, lanes 3 and 6; 30×, lanes 4 and 7; 50×, lanes 5 and 8). (F) Lef1 and β-catenin form complexes on the TCF/LEF site (lane 2). In-vitro-translated His-tagged mLef1 (0.5 μl) and flag-tagged hβ-catenin (4 μl) were used. α-His antibody (2 μl) and α-flag antibody (2 μl) super-shift the complex (lanes 3 and 4). (G) Non-labeled TCF/LEF oligo blocked binding of Lef1 and β-catenin to the labeled probe (lanes 3–5). Non-labeled TCF/LEF mut oligo weakly affected the binding (lanes 6–8). The same amounts of unlabeled oligonucleotides as (E) were used. (H) ChIP-qPCR analysis on chromatin preparations from E9.5–10.0 forelimb-level trunk explants. Primers that amplify a region containing the TCF/LEF site of intron2 were used. A region of exon2 was used as a control. Mean ± SEM; quantification in triplicate; (^∗∗^) p < 0.01 with Student’s t test. Experiments were performed twice, and representative data are shown.

To test whether RA directly controls *Tbx5* transcription, we analyzed the mouse *Tbx5* forelimb regulatory element we have previously identified (Minguillon et al., 2012). The core 361-bp sequence contains putative RAREs and a TCF/LEF-binding site (Figures 4A and 5A) in addition to the Hox-binding sites previously identified (Minguillon et al., 2012; Nishimoto et al., 2014).

A canonical RARE is composed of two repeats of hexameric motifs (G/A)G(G/T)TCA separated by one, two, or five nucleotides (Bastien and Rochette-Egly, 2004; Umesono et al., 1991). RAREs are occupied by RAR/RXR heterodimers constitutively regardless of their ligand-binding state. In the absence of RA, RAR/RXRs are associated with co-repressors and, upon ligand binding, co-activators are recruited to RAR/RXR. In the *Tbx5* enhancer element, there are two sequences similar to the canonical RAREs (RARE2 and 3) and two putative half-sites (RARE half-sites 1 and 4; Figure 4A). To test their function, we generated transient transgenic mice harboring *LacZ* reporter gene under control of the *Tbx5* enhancer element. We chose one of the canonical RAREs (RARE3) and one of the half-sites (RARE1) for further analysis. The 361-bp regulatory element is sufficient for forelimb expression (Minguillon et al., 2012; Figure 4B). Mutation of RARE3 completely inactivates the enhancer, and no expression of the reporter gene was observed in the forelimb bud (Figure 4C). Mutation of the RARE half-site 1 caused reduced expression of *LacZ* with residual expression in the anterior forelimb bud (Figure 4D), probably because this is a half-site. Because mutation of RARE3 caused a dramatic reduction of *LacZ* expression, we tested its ability to bind RAR and RXR in EMSA (Figures 4F and 4G). mRARα and mRXRα form a complex with an oligo containing RARE3 (Figure 4F, lane 3). The specificity of binding was confirmed using an α-flag antibody that recognizes the epitope present in the N-terminal of mRARα (Figure 4F, lane 4) and α-myc antibody that recognizes the epitope in the N-terminal of mRXRα (Figure 4F, lane 5). These proteins fail to form a complex with mutated RARE3 (Figure 4F, lanes 6–10). The RARα-RXRα-oligo complex was abolished by addition of non-labeled competitor (Figure 4G, lanes 2–5), whereas an RARE3-mutated oligo did not affect the complex (Figure 4G, lanes 6–8). These results demonstrate that RARα and RXRα can occupy RARE3 in vitro and indicate this site is bound by retinoid receptors in vivo.

### β-Catenin/TCF/LEF Directly Regulate *Tbx5* Expression

Because the *Tbx5* enhancer element also contains a putative TCF/LEF site (Figure 5A), we tested its requirement for *Tbx5* expression. Wnt secreted by the AER is required for limb outgrowth (Kengaku et al., 1998), and experiments in zebrafish and chick embryos have evoked a Wnt signal regulating limb initiation by controlling *Tbx5* expression (Kawakami et al., 2001; Ng et al., 2002). Its function in mouse limb initiation is unclear because no candidate Wnt ligand has been shown to be expressed at early limb initiation stages and the *Tcf1/Lef1* knockout is able to form limb buds (Galceran et al., 1999). Mutation of the putative TCF/LEF site within the *Tbx5* forelimb regulatory element caused reduced expression of the reporter gene (Figures 5B and 5C). Expression is absent in the forelimb bud whereas some residual expression remains in the LPM both rostral and caudal to the forelimb bud (Figure 5C, arrows). To confirm that the TCF/LEF site can bind TCF/LEF protein, we performed EMSA assays (Figures 5D and 5E). mLef1 can form a complex with an oligo containing the TCF/LEF site (Figure 5D, lane 2), and addition of an α-His antibody that recognizes a His epitope in the N-terminal of mLef1 super-shifted the complex, demonstrating this complex contains mLef1 (Figure 5D, lane 3). An oligo in which the TCF/LEF site is mutated (mut TCF/LEF) has less affinity to mLef1 (Figure 5D, lanes 4–6). Competition assays with unlabeled oligos confirmed the specificity of the complex (Figure 5E). As transcriptional activity of Lef1 is controlled by recruitment of its co-activator β-catenin, we tested whether Lef1 and β-catenin can form a ternary complex on the TCF/LEF site (Figures 5F and 5G). In the presence of β-catenin, an additional slower-migrating band was observed (Figure 5F, lane 2). Both an α-His antibody that recognizes a His epitope of recombinant His-mLef1 protein and an α-flag antibody that recognizes a flag epitope of flag-hβ-catenin protein caused a super-shift of the mLef1-hβ-catenin-oligo complex (Figure 5F, lanes 3 and 4). The oligo-Lef1-β-catenin complex was abolished with non-labeled competitor (Figure 5G, lanes 3–5) whereas the complex was less affected by mutTCF/LEF competitor (Figure 5G, lanes 6–8), demonstrating specificity of the interaction. To test the binding of β-catenin to the *Tbx5* regulatory element in vivo, we carried out ChIP-qPCR analysis of the forelimb-level trunk region from E9.5–10.0 embryos. *Tbx5 intron2* showed an enrichment of binding to α-β-catenin antibody compared to control IgG antibody (p < 0.01; Figure 5H). As a control site, we chose a region in exon2 that we have previously shown does not possess enhancer activity (Minguillon et al., 2012). There was no enrichment in binding in this region with the α-β-catenin antibody compared to the control IgG antibody (Figure 5H).

**Figure 6:**
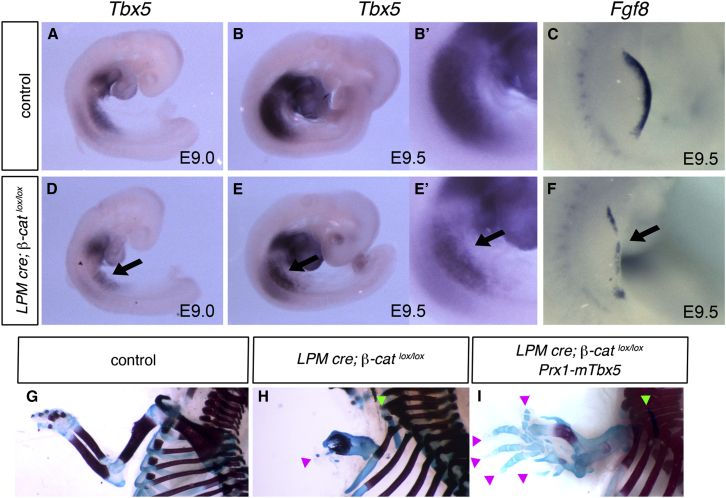
β-Catenin Signal Acts Upstream of *Tbx5* in Mouse Forelimb Initiation (A–F) *WISH* of *Tbx5* (A and B) and *Fgf8* (C) in control embryos. *WISH* of *Tbx5* (D and E) and *Fgf8* expression (F) in *β-catenin* mutant embryos is shown. *Tbx5* expression in the forelimb LPM is reduced in *LPM cre*; *β-catenin*^*lox/lox*^ embryos (D and E, arrows). *Fgf8* expression is downregulated and patchy in mutant forelimb bud (F, arrow). (G–I) Skeletal preparation of control (G), mutant (H), and rescued (I) embryos. *β-catenin* mutant forelimb is shortened and lacks most of digits and scapula (H). Forced expression of *Tbx5* using *Prx1* promoter partially rescues the defects (I). Pink arrowheads indicate the digits, and green arrowheads indicate the scapula remnant.

To test requirement of β-catenin in *Tbx5* expression in vivo, we analyzed β-catenin conditional mutant mice (Huelsken et al., 2001). The limb-bud-restricted *Prx1-Cre* deleter line has been used to conditionally delete β-*catenin* (Hill et al., 2006). *Cre* expression in this strain starts at the 14 somites stage (Hasson et al., 2007), later than the initiation of *Tbx5* expression at the 8 somites stage (Agarwal et al., 2003). Although deletion of *Tbx5* using this *Cre* line produces a forelimb-less phenotype (Rallis et al., 2003), we judged *Cre* expression in this line would not be active early enough to analyze upstream transcriptional regulators of *Tbx5*. We therefore generated a new LPM-specific *Cre* line (*LPMCre*) that is expressed early in the nascent forelimb-forming regions. Intron2 of the *Tbx5* gene with mutations of the second Hox-binding site was used to induce *Cre* expression broadly in the LPM (Nishimoto et al., 2014). *Cre* activity starts by the 10 somite stage (Figure S3A). At E9.5–11.5 there is robust activity in the LPM including the forelimb bud, the inter-limb flank, and the anterior two-thirds of the hindlimb buds, but not in other embryo regions (Figures S3C and S3D). The deletion of β-*catenin* using the *LPMCre* caused reduced expression of *Tbx5* (Figures 6A, 6B, 6D, and 6E), suggesting that β*-catenin* is required for *Tbx5* expression. The residual expression of *Tbx5* in these β*-catenin* mutants can be explained by the timing of the onset of *Cre* expression. *Cre* expression from *LPMCre* is controlled by a *Tbx5*-regulatory element and is active coincident with endogenous *Tbx5* expression; thus, some amount of *Tbx5* is already expressed by the time β-*catenin* is deleted to downregulate *Tbx5* transcription. *Fgf8* expression was reduced in a patchy manner in β-*catenin* mutants, suggesting a defect in AER formation (Figures 6C and 6F). The mutant limbs that develop are severely truncated (Figures 6G and 6H) and lack most of the digits (Figure 6H, pink arrowhead) and scapula (Figure 6H, green arrowhead). As the medial scapular border is somitic in origin (Valasek et al., 2010), the remnant that does form may be derived from these migratory cells. Forced expression of *Tbx5* using a *Prx1* promoter transgenic line, *Prx1-mTbx5*, was able to partially rescue the outgrowth defect (Figure 6I). The rescue was only partial probably because of a wide range of other functions of β-catenin including those in cell adhesion.

To identify Wnt ligands that activate β-catenin/TCF/LEF signaling, we performed in situ hybridization to detect *Wnt2* expression (Figure S4) that had been reported to be expressed in the early mouse embryo flank (Monkley et al., 1996). *Wnt2* is expressed in the LPM at E8.5–9.5, suggesting that *Wnt2* is a potential ligand. Deletion of *Wnt2*, however, does not cause obvious limb defects (Monkley et al., 1996), suggesting that other unidentified Wnt family members have redundant functions with *Wnt2*.

Together, these results suggest that the β-catenin/TCF/LEF pathway promotes forelimb initiation in mouse by direct positive regulation of *Tbx5* transcription.

## Discussion

**Figure 7:**
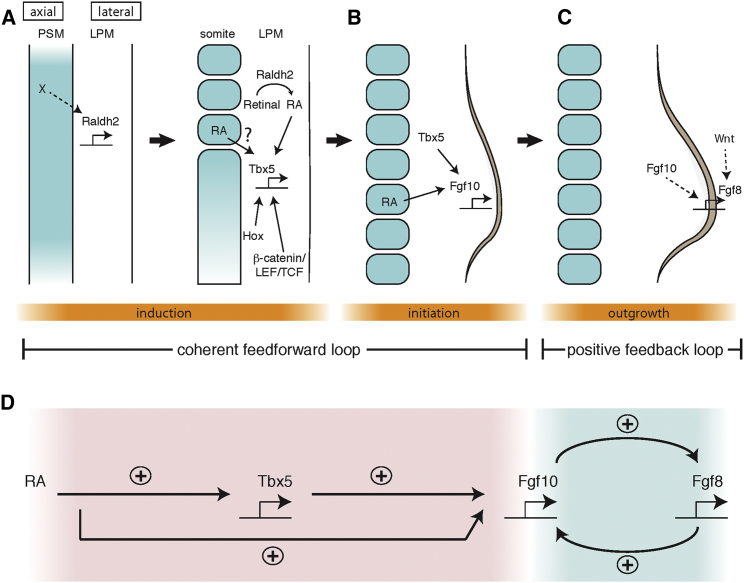
The Molecular Mechanisms of Limb Bud Formation (A) During limb induction, an axial signal is required for *Raldh2* expression in the LPM, which produces RA locally. RA, β-catenin/TCF/LEF, and Hox genes act cooperatively to induce *Tbx5* in the LPM. (B) Subsequently, RA from the somite functions cooperatively with Tbx5 to induce *Fgf10* expression. Thus, RA and *Tbx* transcription factors act in a coherent feed-forward loop (D). (C and D) Fgf10 in limb bud mesenchyme induces *Fgf8* expression (C) in the overlying ectoderm to establish an *Fgf10-Fgf8* positive feedback loop required for outgrowth (D).

In this study, we demonstrate that an RA signal and *Tbx* transcription factors act in a coherent feed-forward loop to establish the positive feedback loop of FGF signaling between LPM and overlying ectoderm to recruit the cohort of progenitors that form the limb bud (Figure 7). At limb induction stages, an RA signal directly activates *Tbx5* transcription, acting cooperatively with β-catenin/LEF/TCF and Hox factors. Subsequently, at limb initiation stages, RA acts cooperatively with Tbx5 in forelimb or Tbx4 in hindlimb to activate *Fgf10* expression. Although we do not know whether RA signaling regulates *Fgf10* directly or indirectly, RA may function as a co-factor of Tbx5 and Tbx4.

### *Tbx5* or *Tbx4* Expression in the LPM Is Not Sufficient to Initiate Limb Outgrowth

Gene deletion experiments in mouse have clearly demonstrated requirement for *Tbx5* and *Tbx4* to establish the Fgf10-Fgf8 positive feedback loop between the limb mesenchyme and overlying ectoderm. Following deletion of *Tbx5*, the forelimb fails to form (Agarwal et al., 2003; Rallis et al., 2003), *Fgf10* expression is not initiated, and the FGF feedback loop is never established. A similar regulatory relationship exists between *Tbx4* and *Fgf10* in the hindlimb; however, there is not an exclusive requirement of *Tbx4* for *Fgf10* to be expressed in the hindlimb because, in *Tbx4* mutants, low levels of *Fgf10* expression are established and a small hindlimb can form (V. Duboc, F. Sulaiman, A. Kucharska, D. Bell, M. Holder-Espinasse, and M.P.O.L., unpublished data; Naiche and Papaioannou, 2003). Additional input from Islet1 and Pitx1 act in the hindlimb to positively regulate *Fgf10*, and these can partially compensate for loss of *Tbx4* (V. Duboc, F. Sulaiman, A. Kucharska, D. Bell, M. Holder-Espinasse, and M.P.O.L., unpublished data; Kawakami et al., 2011; Narkis et al., 2012). Ectopic expression of *Tbx5* or *Tbx4* in the inter-limb flank region of chick embryos is sufficient to induce ectopic limbs (Ng et al., 2002; Takeuchi et al., 2003). Significantly, our results show that, following barrier insertion at stages 12–15, *Tbx5* and *Tbx4* are still expressed in the forelimb- and hindlimb-forming LPM with normal spatial and temporal dynamics. Despite this, *Fgf10* expression is not induced, *Fgf8* expression is not established in the overlying ectoderm, and initiation of limb bud formation fails. This demonstrates that *Tbx5* and *Tbx4* expression in the presumptive forelimb and hindlimb, respectively, is not sufficient to induce limb outgrowth. Because *Tbx5* and *Tbx4* are thought to act directly to regulate *Fgf10*, these results suggest that an additional factor(s) are required to initiate *Fgf10* expression, and we identified RA as such a factor (Figure 2). The apparent sufficiency of *Tbx5* and *Tbx4* to induce ectopic limbs in the inter-limb LPM may be explained by the sustained local supply of RA in the region. *Raldh2* expression remains in the inter-limb LPM after its expression level is reduced and restricted to proximal parts of the limb buds. Thus, RA from the adjacent paraxial mesoderm is required in the limb buds, whereas, in the inter-limb LPM, locally produced RA may be sufficient for ectopically expressed Tbx5 or Tbx4 to form an extra limb.

### The β-Catenin Pathway Is Required for Forelimb Initiation

The requirement for Wnt signaling in limb induction has been demonstrated in chick and zebrafish embryos (Kawakami et al., 2001; Ng et al., 2002). Downregulation of Wnt signaling causes downregulation of *Tbx5* expression, suggesting the Wnt/β-catenin signal acts upstream of *Tbx5*. In mouse, however, the role of Wnt signaling in limb induction is less clear. *Wnt* ligands that are expressed in the LPM or adjacent tissues at these stages have not been identified. In addition, the double knockout mice of *Lef1* and *Tcf1*, two genes from the TCF/LEF family strongly expressed in limb buds, form limb buds (Galceran et al., 1999). In this study, using a novel early acting LPM-restricted cre deleter transgenic (*LPMCre*), we have conditionally deleted *β-catenin* in the forelimb-forming regions and demonstrate that this causes reduced expression of *Tbx5* and a failure to establish normal levels of FGF signaling, ultimately resulting in a truncated forelimb. Conditional *β-catenin* mutant mice have been generated previously using the *Prx1-Cre* or *Hoxb6-Cre* deleter transgenics (Hill et al., 2006; Kawakami et al., 2011). In both examples, *Cre* is not expressed early enough or in the right place to analyze its role in regulating *Tbx5* expression. Thus, we generated a new LPM-specific *Cre* strain and demonstrate that deletion of *β-catenin* leads to reduced expression of *Tbx5* and *Fgf8*, resulting in truncated forelimbs (Figure 6). The forelimb outgrowth defect in *β-catenin* mutant mice was partially rescued by forced expression of *Tbx5* (Figure 6). Our analysis of the *Tbx5* regulatory element reveals that β-catenin signaling regulates *Tbx5* expression directly. These results suggest control of *Tbx5* by β-catenin signaling is conserved in zebrafish, chick, and mouse forelimb initiation. The question still remains how the β-catenin signal is activated. We show that *Wnt2* is a potential ligand as it is expressed in the LPM at E8.5; however, no limb phenotype has been described in the *Wnt2* knockout mouse (Monkley et al., 1996), suggesting that other *Wnt* family members are also involved. No other *Wnt* ligands have been reported to be expressed in the forelimb LPM or adjacent tissues at the limb induction stages; however, it is possible that the expression levels of *Wnt* ligands are below the level of detection by in situ hybridization. Limb bud formation in *Lef1/Tcf1* double knockout mice may be explained by redundant functions between LEF/TCF family genes. Low-level expressions of other *Tcf* genes may be sufficient for limb induction to occur.

### Multiple Signal Inputs Regulate *Tbx5* Transcription in the LPM

Together with our previous studies (Minguillon et al., 2012; Nishimoto et al., 2014), we reveal the spatial and temporal regulatory mechanisms of *Tbx5* expression. In the forelimb-forming region, the positive inputs including Wnt/β-catenin signal, RA signal, and Hox4 and 5 paralogs work together to activate *Tbx5* transcription. *Raldh2* is expressed broadly in the LPM and somites, indicating that RA signal acts as a permissive factor rather than an inducing factor. In competent cells, Hox4/5 and β-catenin signal initiate forelimb progenitor fates and control *Tbx5* expression (Minguillon et al., 2012). *Tbx5* expression is limited to forelimb-forming LPM, and this spatial restriction is achieved by the transcriptional repression by caudal Hox genes such as Hox8, 9, and 10 paralogs (Nishimoto et al., 2014).

Our analysis of the forelimb-regulatory element of *Tbx5* suggests that inputs from RA, β-catenin signaling, and Hox4 and 5 paralogs are all required for normal expression of *Tbx5*, because mutations of either of these sites downregulate the activity of this fragment (Figures 4C, 4D, and 5C; Minguillon et al., 2012; Nishimoto et al., 2014). Following a barrier insertion at stage 9, application of exogenous RA is sufficient to rescue the expression of *Tbx5* (Figures 3B and 3E). This is consistent with results showing that *Wnt-2b* and at least some Hox4 and 5 paralogs are already expressed in the LPM by stage 9 (Barak et al., 2012; Kawakami et al., 2001) and indicates that an axial signal may not be required for expression of these genes.

### An RA Signal Is Required for Limb Induction and Initiation

A requirement for RA signaling in limb formation has been demonstrated by studies of *Raldh2* mutants in zebrafish and mouse (Begemann et al., 2001; Grandel and Brand, 2011; Mic et al., 2002, 2004; Niederreither et al., 1999, 2002). The *Raldh2* mutants lack pectoral fins and forelimb buds in zebrafish and mouse embryos, respectively, and fail to express *Tbx5*. A recent study suggests that RA signaling regulates *Tbx5* indirectly by repressing expression of *Fgf8* in the intermediate mesoderm, heart field, and caudal progenitor zone that can negatively regulate *Tbx5* expression (Cunningham et al., 2013; Zhao et al., 2009). Our results provide evidence for direct regulation of *Tbx5* by RA signaling. Exogenous RA is sufficient to rescue *Tbx5* expression following barrier insertion at stage 9 (Figure 3). In addition, we identify RAREs in the forelimb-regulatory element of *Tbx5* and demonstrate their requirement for activity of the regulatory sequence (Figure 4). Barrier insertion at stage 9 blocks initiation of *Tbx5* expression (Figure 3), but *Tbx5* is expressed following barrier placement later at stage 13 (Figure 1). This indicates that an RA input is only required transiently to establish *Tbx5* expression and is not necessary to maintain *Tbx5* expression. We further demonstrate that RA acts cooperatively with *Tbx5* to activate *Fgf10* expression and as a consequence establish the positive feed-back loop of Fgf10-Fgf8 signaling between the limb mesenchyme and ectoderm. The study of Zhao et al. (2009) also suggests that RA is not required for hindlimb budding. In contrast, our results demonstrate that, in the hindlimb as in the forelimb, application of RA can rescue outgrowth defects in the absence of axial signals (Figures 1 and 2). Insertion of a barrier between the paraxial mesoderm and the LPM prevents the establishment of the Fgf10-Fgf8 feedback loop in both forelimb and hindlimb LPM, and as a result, limb buds are not formed, confirming that an axial signal is required for limb outgrowth. Addition of an RA bead is sufficient to establish the FGF feedback loop and rescue limb formation. Furthermore, an RAR inverse agonist BMS 493 downregulates *Tbx4* in hindlimb induction (Figure 3) and *Fgf10* in hindlimb initiation (Figure 2). These results implicate a key role for RA in limb induction and initiation in both forelimb and hindlimb.

A temporal requirement for RA signaling in limb bud formation has been demonstrated in zebrafish (Grandel and Brand, 2011). An RA signal determines limb precursor cells to express *Tbx5* at gastrula stages, and the same signal later maintains these precursors at somitogenesis stages. The earliest stage we could place a barrier in chick embryos was at presomitic stage, and therefore, we could not examine any potential effects at gastrulation stages. Interestingly, inhibition of RA signal at early somitogenesis stages produces fish that lack pectoral fin buds but express *Tbx5* at the usual rostro-caudal level (Grandel and Brand, 2011), similar to our observation in the chick after late barrier insertion. This suggests that RA may also act cooperatively with Tbx5 in zebrafish pectoral fin formation.

The expression of *Raldh2* in the chick forelimb LPM prior to *Tbx5* suggests that the source of RA at limb induction stages is the LPM in this species (Swindell et al., 1999). Our results demonstrate that this *Raldh2* expression in the LPM requires a signal from axial tissues (Figure 3). In mouse embryos, however, the expression of *Raldh2* in forelimb LPM at pre-limb bud stages has not been reported (Niederreither et al., 1997). Although we cannot exclude the possibility that *Raldh2* is weakly expressed in forelimb LPM and it was not detected, in mouse, the initial source of RA may be neighboring paraxial mesoderm tissues such as the somites that are known to produce RA.

### Conclusions

Here, together with previous studies (Minguillon et al., 2012; Nishimoto et al., 2014), we identify the regulatory network that controls limb induction and initiation. We demonstrate that two key signaling pathways, namely RA and β-catenin pathways, are integrated with positional information determined by nested Hox expression to regulate *Tbx5* expression. Subsequently, Tbx5 acts cooperatively with RA to regulate *Fgf10* expression and initiates the limb outgrowth program. Thus, our results clarify the role of the key regulators of limb bud induction and initiation.

## Experimental Procedures

### Barrier Insertion to Chick Embryos

Fertilized chicken eggs (Henry Stewart Winter Egg Farm) were incubated at 38°C and staged according to Hamburger and Hamilton (1951). To make barriers (0.7–1.3 mm wide), aluminum foil was cut with a scalpel and bent to form a hinge shape using forceps (Stephens and McNulty, 1981; Strecker and Stephens, 1983). Using a tungsten needle, a cut was made through the vitelline membrane and the LPM adjacent to the somites. The barrier was inserted into the cut with forceps (Figures 1A and 1F). The egg was resealed with clear tape and returned to the incubator. Twenty-four hours later, the position of the barrier was noted. Embryos were harvested and fixed in 4% paraformaldehyde (PFA) between 24 and 72 hr after barrier insertion.

### FGF4-, RA-, and BMS-493-Soaked Beads

Affi-Gel Blue beads (Bio-Rad) were rinsed in PBS and then soaked in a 30-μl drop of 0.35 mg/ml FGF4 protein (a gift from Cliff Tabin) on ice for 30 min. Beads approximately 150 μm in diameter were inserted into the cut face of the LPM on the distal side of the incision. The barrier was then inserted proximal to the bead. The same method was used for wing and leg level operations. AG1-X2 formate form ion exchange resin beads (Bio-Rad) were soaked in 0.05–0.1 mg/ml of all-trans-RA (Sigma) diluted in DMSO (Eichele et al., 1984) or 2.5–5.0 mg/ml of BMS 493 (Sigma) diluted in DMSO. 100-μm-diameter beads were inserted using forceps.

### WISH

Whole-mount in situ hybridization (WISH) was carried out essentially as described (Riddle et al., 1993). Full-length cDNA of *mWnt2* was amplified and used as a probe template. The other RNA probes have been described previously: *cTbx5* and *cTbx4* (Logan et al., 1998); *cShh* (Riddle et al., 1993); *cFgf8* (Vogel et al., 1996); *cFgf10* (Ohuchi et al., 1997); *mTbx5* (Rallis et al., 2003); and *mFgf8* (Crossley and Martin, 1995).

### Transient Transgenic Analysis

Transgenic embryos were generated by the Procedural Service section, NIMR by standard pronuclear microinjection techniques. Mouse work was carried out under an appropriate ASPA license granted by the UK Home Office and was subject to local ethical review as outlined in UK Home Office guidelines. Mouse embryos were staged according to Kaufman (2001). Noon on the day a vaginal plug was observed was taken to be E0.5 days of development. Primers and mutated RARE and TCF/LEF sites are listed in Supplemental Experimental Procedures.

### Histology

The cartilage and bone elements of mouse embryos and newborn pups were stained with Alcian Blue and Alizarin Red, respectively, essentially as described previously (McLeod, 1980).

### Electrophoretic Mobility Shift Assays

In-vitro-translated proteins were produced using a TnT Coupled Reticulocyte Lysate System (Promega). Proteins were labeled with 35^S^-Methionine (PerkinElmer) to verify and quantify translation. EMSA was carried out essentially as previously described (Forman et al., 1992; Shtutman et al., 1999). Antibodies and probe sequences are listed in Supplemental Experimental Procedures.

### ChIP-qPCR

Chromatin immunoprecipitation was performed using a previously published method (Tee et al., 2014) with some modifications (see Supplemental Experimental Procedures).

## Author Contributions

S.N., S.M.W., and M.P.O.L. designed the experiments, analyzed the results, and wrote the paper. S.N. and S.M.W. carried out the experiments. S.W. generated the transgenic mouse lines.

## Supplemental Information

Document S1. Supplemental Experimental Procedures, Figures S1–S4, and Tables S1–S3

Document S2. Article plus Supplemental Information
