# Supplementary material for: RA Acts in a Coherent Feed-Forward Mechanism with Tbx5 to Control Limb Bud Induction and Initiation
Source: Cell Rep. 2015 Jul 23;12(5):879–91. doi: 10.1016/j.celrep.2015.06.068 (PMC4553633; doi:10.1016/j.celrep.2015.06.068)
Supplement: Document S1. Supplemental Experimental Procedures, Figures S1–S4, and Tables S1–S3 [file mmc1.pdf]

Cell Reports

Supplemental Information

RA Acts in a Coherent Feed-Forward Mechanism with *Tbx5* to Control Limb Bud Induction and Initiation

Satoko Nishimoto, Susan M. Wilde, Sophie Wood, and Malcolm P.O. Logan

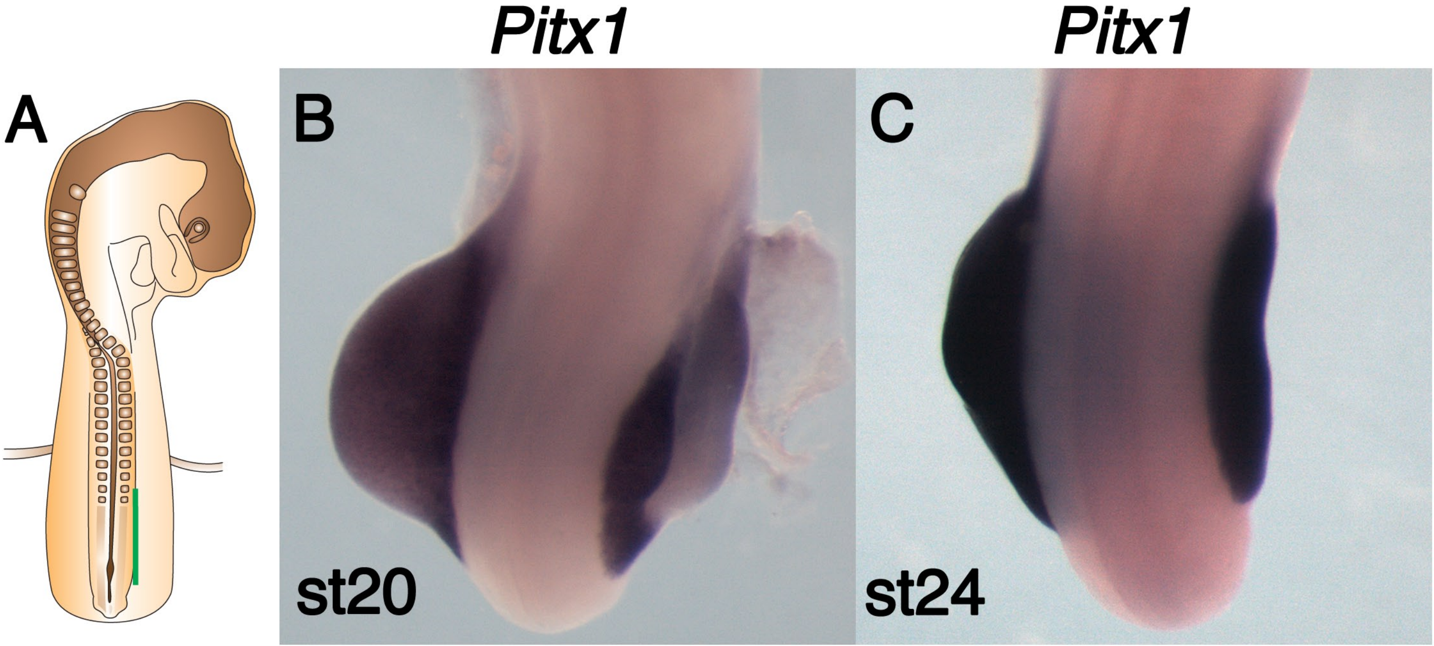

Figure S1, Related to Figure1. Barrier insertion at the leg forming region does not affect *Pitx1* expression.
A. Schematic diagram showing barrier position (indicated by the green line) between the somites or presumptive somites and the LPM at the presumptive leg level (somites 26-32) in stage 15 embryos. **B-C.** Whole mount *in situ* hybridisation analysis on operated embryos. **B**. *Pitx1* is expressed in the LPM at the same rostro-caudal level as the control left leg bud. **C**. *Pitx1* expression is maintained in the right leg region at stage 24 despite absence of limb outgrowth.

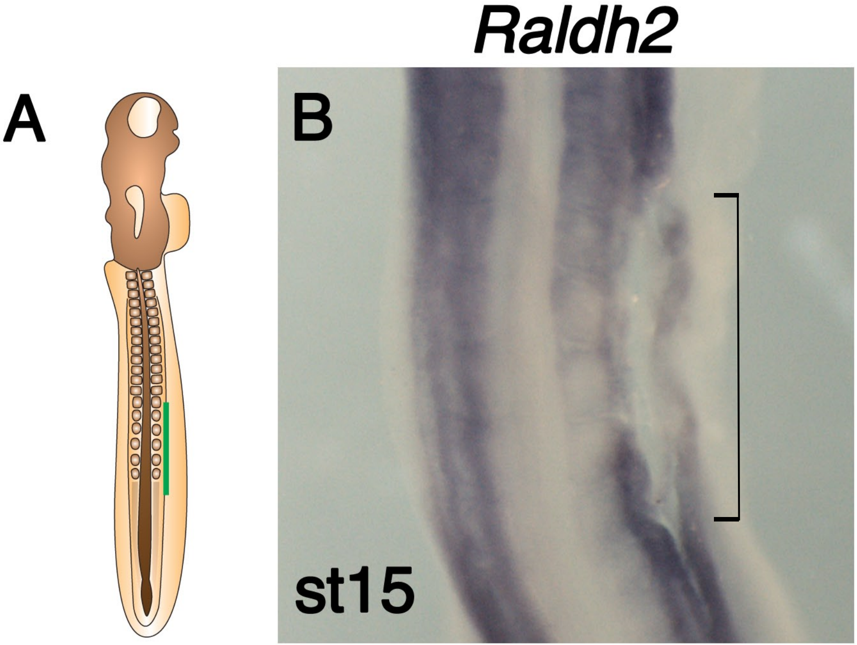

Figure S2, Related to Figure2. Barrier insertion at the forelimb forming region does not affect *Raldh2* expression. **A**. Schematic diagram showing barrier position (indicated by the green line) between the somites and the LPM at the presumptive forelimb level (somites 15-20) in stage 13 embryos. **B.** Whole mount *in situ* hybridisation analysis on operated embryos. *Raldh2* is expressed in the LPM distal to the barrior on the right side (bracket).

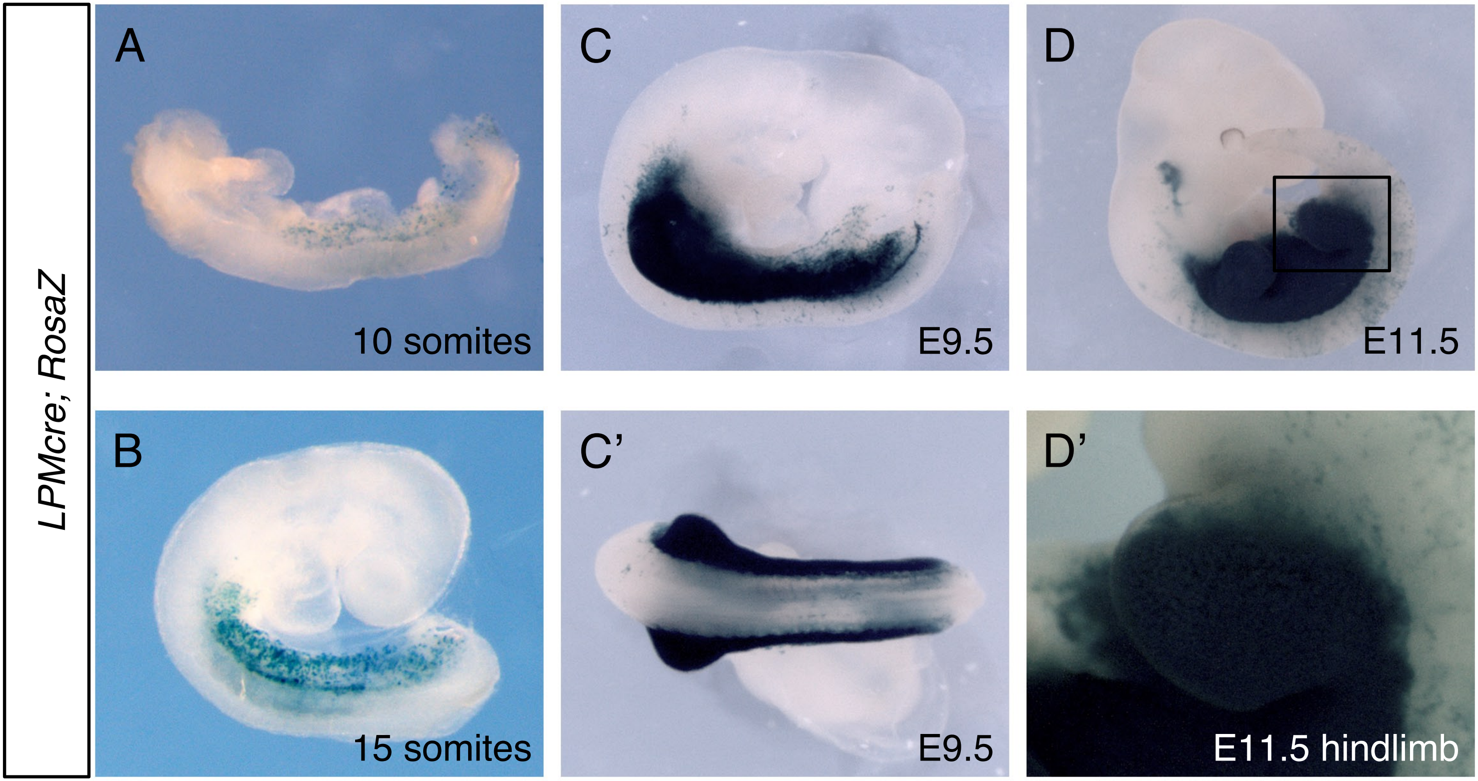

Figure S3, Related to Figure 6. Analysis of Cre recombinase activity of LPMcre.
A-D. *Rosa26RlacZ* reporter line was used. Embryos at 10 somites stage (A), 15 somites stage (B), E9.5 (C-C') and E11.5 (D-D') were stained for β-galactosidase. Cre activity was detected at the10 somite stage (A) and at E9.5 there was robust activity broadly in the LPM (C-C'). The expression domain covers the forelimb bud, the inter-limb flank and the anterior two thirds of the hindlimb (D-D'). **D'**. Hindlimb of the embryo shown in D at higher magnification.

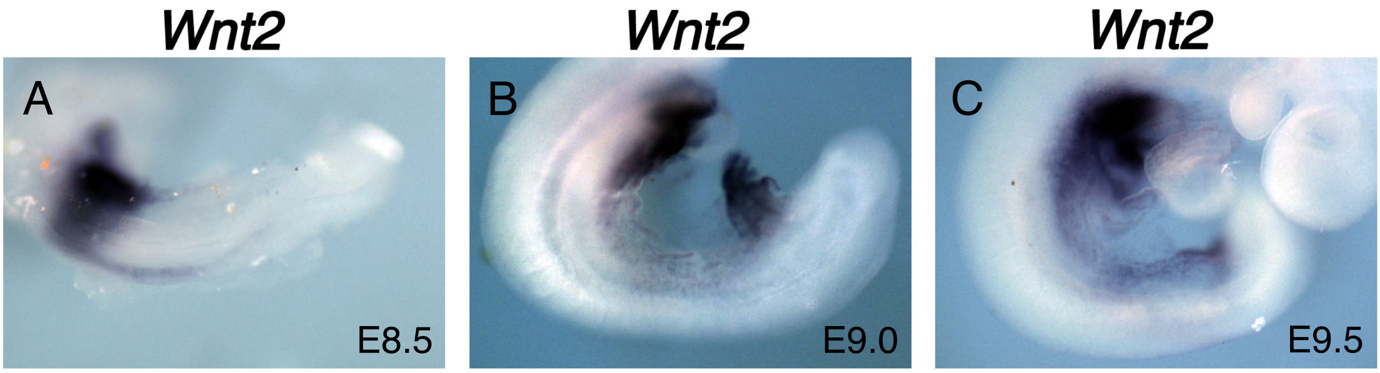

Figure S4, Related to Figure 6. Whole mount *in situ* hybridization showing an expression of *Wnt2* in the LPM. (A) Dorsal view of E8.5 embryo. Its expression is restricted to the rostral LPM. (B and C) Lateral views of E9.0 (B) and E9.5 (C) embryos. *Wnt2* is expressed in the inter-limb LPM and the LPM ventral to limb buds at these stages.

Table S1, Related to Figure 1-3 Wing Level Barrier Experiments

Stage barrier placed (+ bead) *	Number operated	Wing missing **	Wing present **	Number dead
Stage 12-14	40	24	1	15
Stage 8-9	65	23	0	42
Stage 12-13 (FGF4 bead)	41	7 (bead missing)	17	17
Stage 12-13 (RA bead)	75	9 (bead missing)	32	34
Stage 13 (control bead)	5	3	0	2

* 0.7-1 mm foil barriers placed between somites 15-20 and the lateral plate mesoderm. 0.35 mg/ml FGF4 bead. 0.05-0.1 mg/ml RA bead. DMSO control bead.

**Chick embryos fixed stages 21-23

Table S2, Related to Figure 1-3 Leg Level Barrier Experiments

Stage barrier placed (+ bead) *	Number operated	Leg missing **	Leg present **	Number dead
Stage 12-15	43	39	0	4
Stage 10-11	23	16	0	7
Stage 15 (FGF4 bead)	8	0	5	3
Stage 15 (RA bead)	18	0	18	0

* 1.2-1.3 mm foil barriers placed between somites 26-32 and the lateral plate mesoderm. 0.35 mg/ml FGF4 bead. 0.05-0.1 mg/ml RA bead.

**Chick embryos fixed stages 18-24

Table S3, Related to Figure 2 Leg Level BMS493 Bead Experiments

Number operated	Leg bud small	Leg bud slightly small	Leg bud normal size	Number dead
BMS493 beads *				
15	12	3	0	0
DMSO beads**				
12	0	3	9	0

*2-3 beads soaked in 5mg/ml BMS493 placed in the right stage 14-15 LPM at leg level.

**2-3 beads soaked in DMSO alone placed in the right stage 15 LPM at leg level.

All embryos fixed at stage 17-19.

Supplemental Experimental procedures

Barrier insertion to chick embryos

On fixation barriers were removed to prevent them from damaging the embryos during the *in situ* hybridisation process. Barriers of varying widths (measured using a stage graticule) were inserted into embryos between stages 8 and 15 at different rostro–caudal positions along the lateral plate mesoderm at its junction with axial tissues.

Transient transgenic analysis

Mice carrying the *LacZ* transgene were identified by PCR using specific primers (*LacZfwd*, 5'GGTCGGCTTACGGCGGTGATTT3'; *LacZrev*, 5'AGCGGCGTCAGCAGTTGTTTTT3').

Sequences surrounding RAREs and TCF/LEF binding site and the mutations induced are as follows: RARE1, TTTG**GGGTCA**GCTA; RARE1 mut, TTTG**GGGACG**GCTA; RARE3, TAAGGA**GGGTCA**AGTCAT**CGCTCA**TGCCGG; RARE3 mut, TAAGGA**GGGAGC**AGTCAT**CGCAGC**TGCCGG; Tcf/Lef, CTAAGAAAG**ATCTAAG**GAGGGTCAA; Tcf/Lef mut, CTAAGAAAG**ATGTGAG**GAGGGTCAA.

Electrophoretic Mobility Shift Assays

Antobodies recognizing myc epitope (Santa Cruz sc-40), His epitope (invitrogen R940-25) and flag epitope (Sigma, F3165) were used. Probe sequences used are as follows: RARE3, CCGGCATGAGCGATGACTTGACCCTCCTTA; RARE3 mut, CCGGCAGCTGCGATGACTGCTCCCTCCTTA; Tcf/Lef,

TTGACCCTCCTTAGATCTTTCTTAG; Tcf/Lef mut, TTGACCCTCCTCACATCTTTCTTAG.

ChIP-qPCR

Trunk tissues at forelimb level of E9.5-10.0 mouse embryos were fixed in 2 mM Di(N-succinimidyl) glutarate for 45 minutes, homogenized with 23G and 25G needles and fixed further in 1 % formaldehyde. Cells were resuspended in buffer containing 50 mM HEPES (pH 7.5), 140 mM NaCl, 1 mM EDTA, 10% glycerol, 0.5% NP-40, 0.25% Triton X-100 and *cOmplete Protease Inhibitor* Cocktail (Roche) and rocked at 4°C for 10 minutes. Cells were collected and resuspended in 200 mM NaCl, 1 mM EDTA, 0.5 mM EGTA, 10 mM Tris (pH 8) and protease inhibitors and incubated for 10 minutes at room temperature to extract nuclei. Nuclei were resuspended in sonication buffer (1 mM EDTA, 0.5 mM EGTA, 0.5% N-lauroylsarcosine, 10 mM Tris at pH 8 and protease inhibitors) and sonicated 30s ON/OFF for 20 minutes in a Bioruptor sonicator to length of 200-500 bp. 50 μg of chromatin was incubated with 0.3 μg of each antibody (IgG, DAKO Z0259; α-β-catenin antibody, Santa Cruz sc-7199) in buffer containing 5.67 mM EDTA, 0.33 mM EGTA, 0.33% N-lauroylsarcosine, 1% Triton X-100, 0.1% sodium deoxycholate, 6.7 mM Tris at pH 8 and protease inhibitors at 4°C overnight. Next, 15 μl of Dynabeads (Invitrogen) was added and incubated at 4°C for 2 hours. Immunoprecipitates were washed five times with RIPA buffer (0.25 m LiCl, 50 mM HEPES pH 7.5, 1 mM EDTA, 1% NP-40, 0.7% DOC, and protease inhibitors). Pellets were resuspended in 100 μl of TE and 5 μl was used for qPCR analysis. Primer sequences used are as follows: intron2 fwd, CCTGGGTATGCCTTATTAAGGGTC;

intron2 rev, AGGCGAGCCGAGGCGAAG; exon2 fwd, ACCATGGCCGATACAGATGA; exon2 rev, GGGATGATGGAGACTTGCTG.
